# Cerebral Responses to Acupuncture at GV24 and Bilateral GB13 in Rat Models of Alzheimer's Disease

**DOI:** 10.1155/2018/8740284

**Published:** 2018-04-30

**Authors:** Shaoyang Cui, Mingzhu Xu, Jianting Huang, Qing Mei Wang, Xinsheng Lai, Binbin Nie, Baoci Shan, Xun Luo, John Wong, Chunzhi Tang

**Affiliations:** ^1^Department of Rehabilitation, Shenzhen Hospital of Guangzhou University of Chinese Medicine, Shenzhen, China; ^2^Stroke Biological Recovery Laboratory, Spaulding Rehabilitation Hospital, Harvard Medical School, Charlestown, MA 02129, USA; ^3^Clinical Medical College of Acupuncture and Rehabilitation, Guangzhou University of Chinese Medicine, Guangzhou, China; ^4^Key Laboratory of Nuclear Analysis Techniques, Beijing Engineering Research Center of Radiographic Techniques and Equipment, Institute of High Energy Physics, Chinese Academy of Sciences, Beijing, China; ^5^Kerry Rehabilitation Medicine Research Institute, Shenzhen, China; ^6^School of Nursing, MGH Institute of Health Professions, Boston, MA 02129, USA; ^7^Department of Occupational Therapy, MGH Institute of Health Professions, Boston, MA 02129, USA

## Abstract

Acupuncture has been widely used in China to treat neurological diseases including Alzheimer's disease (AD). However, its mechanism remains unclear. In the present study, eighty healthy Wistar rats were divided into a normal control group (*n* = 15) and premodel group (*n* = 65). Forty-five rats that met the criteria for the AD model were then randomly divided into the model group (MG), the nonacupoint group (NG), and the acupoint group (AG). All rats received positron emission tomography (PET) scanning, and the images were analyzed with Statistical Parametric Mapping 8.0. MG exhibited hypometabolism in the olfactory bulb, insular cortex, orbital cortex, prelimbic cortex, striatum, parietal association cortex, visual cortex, cingulate gyrus, and retrosplenial cortex. AG exhibited prominent and extensive hypermetabolism in the thalamus, hypothalamus, bed nucleus of the stria terminalis, cerebral peduncle, midbrain tegmentum, and pontine tegmentum compared to NG. These results demonstrated that acupuncturing at GV24 and bilateral GB13 acupoints may improve the learning and memory abilities of the AD rats, probably via altering cerebral glucose metabolism (CGM) in the hypothalamus, thalamus, and brain stem. The observed effects of acupuncture may be caused by regulating the distribution of certain kinds of neurotransmitters and enhancing synaptic plasticity.

## 1. Introduction

Alzheimer's disease (AD) is a neurodegenerative brain disease and the most common type of dementia in the elderly [1], accounting for approximately 40–50% of the total cases of dementia [2]. The symptoms of AD inevitably worsen progressively and lead to fatality [3]. At present, over 40 million people worldwide suffer from AD [4], including approximately 700,000 Americans aged 65 years or older in the United States, where it costs $259 billion in 2017 [5].

The main neuropathological features of AD are senile plaques and neurofibrillary tangles [6], which gradually accumulate in the brain. As the disease progresses, these features damage brain circuits involved in cognition. The diagnosis of AD is mainly based on the clinical manifestation of symptoms, including progressive cognitive deficit, initially confined to episodic memory systems [7].

Recently, noninvasive neuroimaging has also been used increasingly in the diagnosis of AD [8], as cerebral glucose metabolism (CGM) is a consistent feature of AD [9]. ^18^F-2-Fluoro-deoxy-D-glucose (^18^F-FDG) positron emission tomography (PET) allows the observation of metabolic processes by monitoring positron-emitting radioactive tracers [7] as a marker of neuronal activity [10] and synaptic density [11]. As previous studies have shown that the metabolic changes of the extensive functional networks of the brain are associated with AD [12], some researchers have accepted FDG-PET as a suitable technique for the early and differential diagnosis of dementia [13], with a sensitivity of 94% and specificity of 73% [14].

Acupuncture has been used as a kind of traditional Chinese medicine (TCM) therapy to treat senile dementia including AD in the past decades. Satisfactory and promising effects have been reported in clinical trials and animal experiments [15–19]. Shenting (GV24) and bilateral Benshen (GB13), belonging to the Governing Vessel and Foot Shaoyang Gall Bladder Channel, respectively, are commonly used in treating memory impairment and cognitive deficits [20–22]. However, the mechanism is uncertain. According to the TCM theory, the therapeutic effect of acupuncture is mainly related to the selected acupoints for acupuncture manipulation [23]. Therefore, the acupoint is a breakthrough in understanding the mechanism of acupuncture.

With the integration of acupuncture and modern imaging techniques, research has been conducted based on PET to observe the brain responses of acupuncture [24–26]. We hypothesized that the efficacy of acupuncture is achieved by stimulating specific acupoints, initially targeting certain parts of the brain and subsequently causing neurohumoral interactions to rebalance body homeostasis. In our study, Shenting (GV24) and bilateral Benshen (GB13) were selected to explore the effects of acupuncture in the brain of a rat model of AD.

## 2. Materials and Methods

### 2.1. Animals

Eighty adult Wistar rats (40 females and 40 males) weighing 200–250 g were obtained from the China Academy of Chinese Medical Sciences. All rats were housed in standard rodent laboratory cages with a temperature of 20 ± 2°C, relative humidity of 55 ± 10%, and lights on from 6 am to 6 pm each day. Sterilized drinking water and standard diet were supplied ad libitum. All rats were allowed to have seven days acclimation before any experimentation. The experimental protocols were in strict accordance with the guidelines of the care and use of laboratory animals of the Ministry of Science and Technology of the People's Republic of China and were approved by the Animals Research Ethics Committee of Guangzhou University of Chinese Medicine (20110032SPF). All efforts were made to minimize the animals' suffering.

### 2.2. Reagents and Instruments

Reagents and instruments used in this study are listed in Table 1.

### 2.3. Modeling Procedures

All rats were randomly divided into a premodel group (PMG) (*n* = 65, including 32 males and 33 females) and a control group (CG) (*n* = 15, including 8 males and 7 females). First, the PMG received intraperitoneal D-galactose injections (10 mg in 1 ml 0.9% saline) each day for 6 weeks. Then after intraperitoneal injection of pentobarbital anesthesia (50 mg/kg), the rat's scalp was cut open to expose the skull and put in a stereotaxic frame. A microinjection needle was placed in the hippocampal CA1 regions, 3.5 mm lateral and 2 mm posterior to the brain bregma and 2.7 mm deep under the skull surface [27]. A*β*1–40 was injected at a rate of 0.4 *μ*l/min. After that, the brain scalp was sutured. Gluteus intramuscular injection of penicillin (40,000 U/day) was performed for three days. At the same time, CG received an intraperitoneal injection of sterile normal saline for 6 weeks. The Y-maze task was used to gauge the success of modeling two weeks after the A*β*1–40 injection. The criteria of the AD model, based on the Y-maze result from the control group, is described below. Rats in PMG that met the criteria of the AD model were included in the next step. Eleven rats (6 females and 5 males) died during D-gal and A*β*1–40 administration. Nine rats (5 females and 4 males) did not meet the model criteria. Therefore, 45 rats (23 males and 22 females) from PMG were included, and they were then randomly divided into three groups: the model group (MG) including 8 females and 7 males, the nonacupoint group (NG) including 8 females and 7 males, and the acupoint group (AG) including 7 females and 8 males. All rats underwent micro-PET scanning.

### 2.4. Acupoint and Nonacupoint Locations

**Figure 1:**
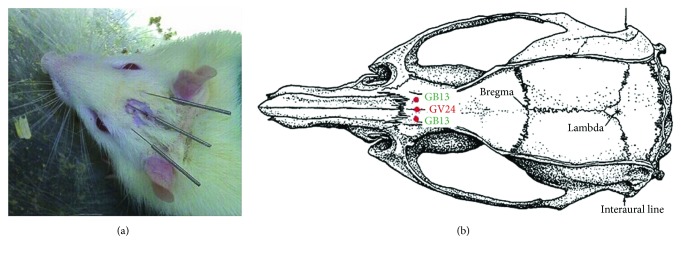
Acupuncture points GV24 and bilateral GB13.

GV24 is located 2 mm directly above the midpoint of the rat's eyes, while GB13 is located 3 mm lateral to GV24, both of which are in the scalp of the frontal pole (Figure 1) [28]. The nonacupoints are located under the bilateral costal arch regions, 10 mm above the crista iliaca, which are not true acupoints and do not belong to any meridians in the theory of TCM.

### 2.5. Acupuncture

Acupuncture manipulation was performed in both AG and NG. Each rat was needled once per day, 5 days a week for a total of 4 weeks. A sterilized, disposable, stainless steel acupuncture needle (25 mm in length and 0.2 mm in diameter) was inserted obliquely as deep as 4-5 mm into the acupoints or the nonacupoints. The needles were mildly twirled, lifted, and thrusted to equally reinforce and reduce. The twisting was performed at a range of 90–180° and a rate of 100–150 times/min. The lifting and thrusting were performed at a range of approximately 2-3 mm and a rate of 60–90 times/min. Lasting about 15 minutes per rat, the manipulation process was performed by the same acupuncturist during the experiment from 8 a.m. to 12 a.m. every day.

### 2.6. Y-Maze Task

Fourteen days after the A*β*1–40 injection, the Y-maze task was conducted in a quiet, dark environment from 2 p.m. to 6 p.m. every day. The task was administered by two specially assigned workers who were blinded to the experimental groups and the interventions. The Y-maze was a dark, opaque plexiglass cage, with a conductive grid floor consisting of three identical arms (I-II-III, 40 L × 25H × 10 W cm each) positioned at equal angles. One light bulb was fixed on top of each arm. The grid floor and light bulbs were connected to an electric controller. The arm of the Y-maze with a light represented the safety zone, while the other dark arms were nonsafety zones where grid floor shocks were applied [29].

### 2.7. Pretreatment and Posttreatment Memory Test

After 5 minutes of adaptation to the Y-maze, rats were trained to learn that the arm with the light was the safe direction by changing the safety zone and the electric shock zone. The electric stimulation parameters were set to 50 V with 5 seconds of delay. If the rat could run to the safety zone directly in 10 seconds after receiving an electric shock on its feet, it was considered the correct reaction; otherwise, it was considered incorrect. The training was repeated 20 times everyday. The pretreatment memory test was performed on day 4, while the posttreatment memory test was performed 4 weeks after acupuncture treatment. The total reaction time (TRT) was recorded to reflect spatial working memory. The test was performed as follows: the safety zone was changed in the order of arm I-II-III-I. The light would be kept on for 10 seconds after the rat ran into the safety zone. After the lights were turned off, one test was completed. After a break of 20–30 seconds, another test would commence. A statistically significant difference between an experimental group and CG was set as the criteria for the AD model. The maze was cleaned with 0.9% sodium chloride solution and dried with a cloth before the next rat was tested.

### 2.8. Brain ^18^F-FDG Micro-PET Imaging

After the Y-maze task, all rats received micro-PET scanning in batches. First, the initial blood glucose level was determined, and the rats were then allowed to rest for 20 minutes in a darkroom. Then ^18^F-FDG (a dose of 0.11 mci per kg) was injected intravenously through the tail vein. After that, they were kept in a quiet environment to allow ^18^F-FDG uptake which lasted about 40 minutes [30]. Then, each rat received a brain scan under anesthesia using 1% isoflurane inhalation. During the scanning, the rat was placed on the scanner bed in a prone position with a plastic stereotactic head holder in place for 10 minutes. ^18^FDG-PET imaging was performed at the PET Center of China PLA General Hospital with a micro-PET/CT imaging system (Explore Vista CT, GE, USA). A 1.0 mm full width at half maximum (FWHM) at the center of the field of view (FOV) was set as the radial spatial resolution. All the images were reconstructed on a 175 × 175 × 61 matrix, with the voxel size equals 0.39 × 0.39 × 0.77 mm through a 3D ordered set expectation maximization (3D-OSEM) algorithm. All scans were saved in the Analyze format.

### 2.9. Statistical Analysis and Image Processing

All Y-maze test data were analyzed using IBM SPSS Statistics 21.0 and presented as mean ± standard deviation. Differences between groups were determined by one-way analysis of variance (ANOVA) using the least significant difference (LSD) method for post hoc tests. Statistical significance referred to *P* value < 0.05.

The PET imaging data analysis was performed using spmratIHEP [31, 32] based on Statistical Parametric Mapping 8.0 (SPM8). First, we removed body tissues and background of rat images by MRIcro, and repositioned the original image at D3V which corresponded to the standard template. Then, data sets were processed automatically in spmratIHEP as follows: (1) Individual brain images were spatially normalized to Paxinos and Watson space, and then the voxel size was scaled up four times in the analysis header, registering to the FDG-PET template, removing extracranial tissues, and shearing matrix to delete background. (2) All the normalized images were smoothed through a Gaussian kernel of 2 × 2 × 4 mm^3^ FWHM. Then based on the framework of the general linear model (GLM), the preprocessed brain images were analyzed. An independent sample *t*-test was used to determine differences in FDG signals between two groups. Finally, the brain regions with significant changes in FDG were valued by a voxel-level height threshold of *P* < 0.001 (uncorrected) and a cluster size ≥ 50 voxels.

## 3. Results

### 3.1. Y-Maze Task

**Figure 2:**
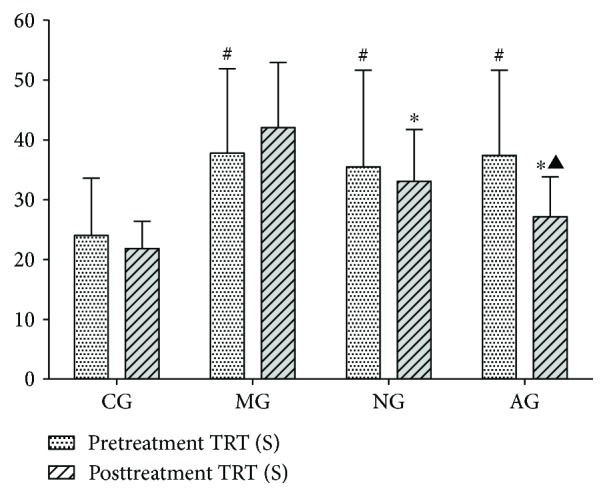
The total reaction time (TRT) of pretreatment memory and posttreatment memory in the Y-maze task. Pretreatment: TRT on the 4th day; posttreatment: TRT on the 29th day. ^#^*P* < 0.05 compared with CG. ^∗^*P* < 0.05 compared with the MG. ^▲^*P* < 0.05 compared with NG.

There was a statistically significant difference in the TRT of pretreatment memory among the different groups (*F*(3,56) = 3.366; *P* = 0.025): the TRT of pretreatment memory was much longer in MG (*P* = 0.028), NG (*P* = 0.026), and AG (*P* = 0.010) compared with CG (Figure 2). After 4 weeks of acupuncture stimulation, there was a statistically significant difference in the TRT of pretreatment memory among the different groups (*F*(3,56) = 17.459; *P* < 0.001): the TRT of posttreatment memory in AG was shorter than that of both MG (*P* < 0.001) and NG (*P* < 0.001).

### 3.2. PET Imaging

**Figure 3:**
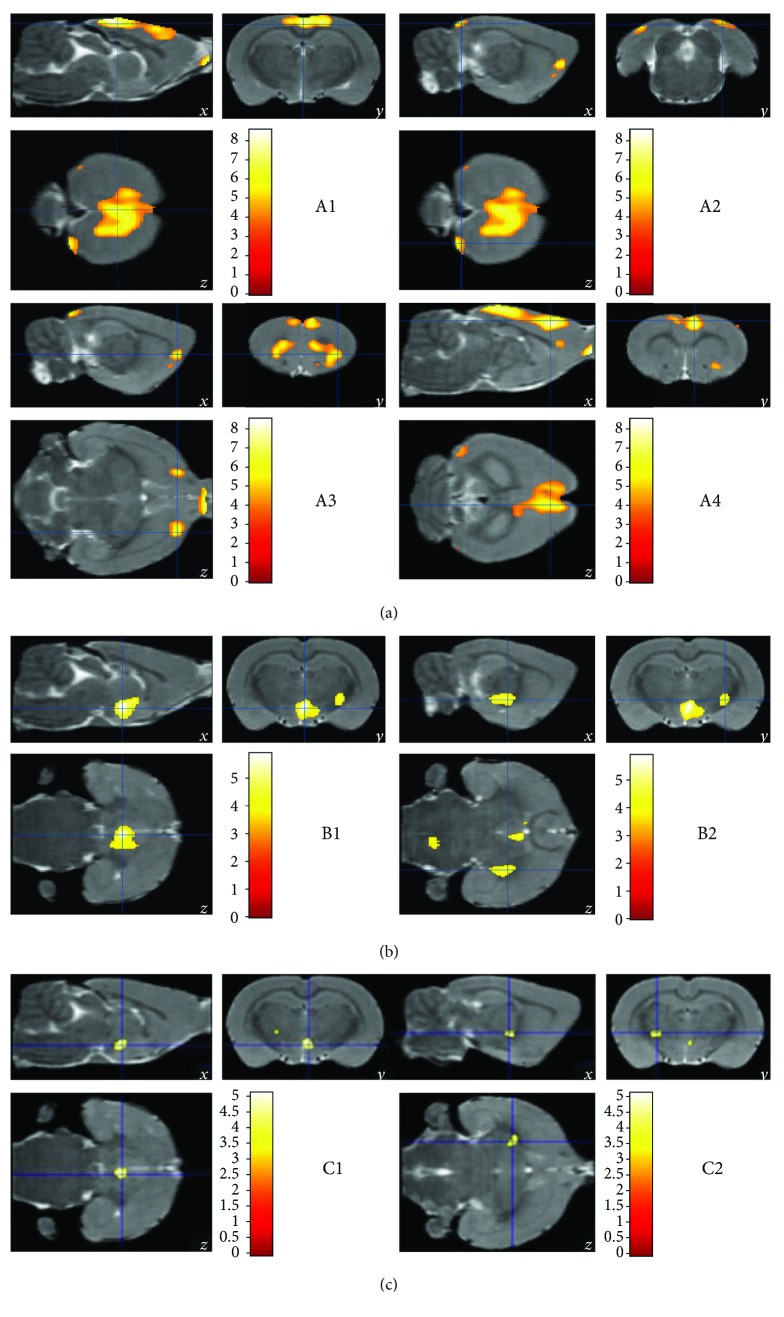
Cerebral areas with decreased (a) or increased (b and c) glucose uptake. The areas with significantly different FDG-PET signals in CG versus MG (a), AG versus MG (b), and NG versus MG (c) (*P* < 0.05) identified in Tables 2, 3, and 4 are shown in these FDG-PET three-dimensional (3D) images. All the data were analyzed with a 2-tailed *t*-test for independent samples.

MG exhibited hypometabolism in the olfactory bulb, insular cortex, orbital cortex, prelimbic cortex, striatum, parietal association cortex, visual cortex, cingulate gyrus, and retrosplenial cortex (Table 2 and Figure 3(a)). AG exhibited hypermetabolism in the bed nucleus of the stria terminalis, lateral and midline nucleus group of the dorsal thalamus, mammillary, supraoptic, and tuberal regions of the hypothalamus, cerebral peduncle, midbrain tegmentum, and pontine tegmentum after 4 weeks (Table 3 and Figure 3(b)). NG showed hypermetabolism in the lateral and midline nucleus group of the dorsal thalamus and mammillary and tuberal regions of the hypothalamus after 4 weeks (Table 4 and Figure 3(c)).

## 4. Discussion

AD begins with impaired memory, gradually leading to total cognitive dysfunction [33]. To date, evidence supports the idea that dysfunction in learning and memory abilities due to structural and functional damage to specific brain regions is associated with AD [34]. It has been demonstrated that hypometabolism, representing synaptic abnormality or neuronal injury, may be also a part of the pathophysiology of AD [14, 35, 36].

In our study, we observed decreased CGM in many areas of the brain, consistent with published findings. People with AD have reduced activity in the retrosplenial cortex [37]. Evidence has shown that the retrosplenial cortex, which is involved in forming and retrieving associations following neutral stimuli, contributes to hippocampal-dependent forms of learning and memory [38], and it connects the hippocampus to the visual cortex and other brain regions. A large amount of amyloid deposits have been found in the retrosplenial cortex of AD patients [39], suggesting that this may be an important area in the development of AD. The parietal association cortex involved with spatial navigation and memory is associated with different sensory modalities [40]. The reduced CGM in this area that we and others have observed may be an important trigger for the onset of AD [41]. Previous studies have reported that AD patients exhibit hypometabolism in the cingulate gyrus from an early stage [42]. The Papez circuit enables the connection between the cingulate gyrus and hippocampus, and it plays an important role in memory function [43]. Recently, the hypometabolism of the olfactory bulb has served as a potential marker of the diagnosis of AD and is related to memory deficits [44, 45]. Although some studies suggested that A*β* deposits in this area may promote AD pathology [46, 47], another study has found that the deposition of the TAR DNA-binding protein of 43 kDa (TDP-43) in the olfactory bulb was associated with AD and associated symptoms including episodic memory loss and hippocampal atrophy [48, 49]. Consistent with the previous finding, metabolic decline was observed in the striatum, probably due to the loss of synaptic contacts [50]. MRI analysis has shown that the atrophy of the striatum may also be involved in the development of AD [51]. Therefore, our rat model could reliably reproduce glucose hypometabolism characteristic of AD, and we speculate that the association between hypometabolism in these brain areas and memory deficits may reflect disease-related dysfunction in these brain regions.

Our results demonstrated that the activated brain regions in AG were much more prominent and extensive compared to NG in AD rats. Taking our Y-maze data into consideration, we speculate that acupuncture at GV24 and bilateral GB13 may improve a memory deficit through activating the thalamus, hypothalamus, and brain stem. The midline group nuclei of the thalamus have close connections with the hippocampus, and previous studies have showed that stimulation of the midline group may activate CA1 and improve acquisition memory and short-term memory by enhancing synaptic plasticity and altering A-beta clearing in rat models of the AD model [52]. The right ventrolateral thalamus has also been demonstrated to contribute more to memory recall while the left side to memory encoding [53]. Recent studies have found volume atrophy and functional degeneration in the thalamus and hippocampus in AD [54, 55]. Neurodegeneration in these structures may lead to further degeneration of the connected regions [56]. It has also been suggested that AD-related memory loss is contributed by the impairment of connections between the anterior thalamus and hippocampus [57]. Lai et al. hypothesized that acupuncture can prevent atrophy and degeneration of AD-related regions by increasing regional glucose metabolism to postpone the progress of the disease and further improve memory and learning abilities [58]. The hypothalamus is highly interconnected with the brainstem and its reticular formation, other limbic structures including the amygdala and septum, and areas of the autonomous nervous system. The hypothalamus can influence the hippocampus-dependent memory function through the limbic response by the activation of the fornix [57]. The brainstem contains many nuclei which are interconnected with the higher cortical or limbic association areas [59]. It regulates cognitive, autonomic, and behavioral functions via its wildly distributed norepinephrine, dopamine, and serotonin neurotransmitter projections to modulate the cortical circuits [60]. Lee et al. postulated that cognitive symptoms such as memory impairment may be related to the morphological alterations of the brainstem in AD patients, as regionally contracted areas were observed in the upper midbrain areas and posterior portion of the brainstem [59].

Acupuncture stimulation may alter brain metabolism by affecting the structure, function, and signaling pathways in the brain. Previous studies have shown that acupuncture on specific acupoints can modulate the cerebral blood flow and strengthen the hippocampal connectivity [61, 62]. Recently, it was also reported that phosphorylated adenosine monophosphate-activated protein kinase (AMPK) and AKT serine/threonine kinase were two critical metabolic homeostatic factors for glucose metabolism, and several studies have demonstrated that acupuncture can activate the expression of AMPK and AKT in rat models of AD [63, 64] and decrease the accumulation of A*β* in the cortex and hippocampus by inhibiting the phosphorylation level of the mammalian target of rapamycin (mTOR) [65]. Other possible effects of acupuncture may also be related to its ability to decrease the level of cytokines in the hippocampus [66], reverse the decreased acetylcholine neurotransmission [67], and reduce oxidative damage by increasing the activity of superoxide dismutase (SOD) [68]. The inhibition of nerve apoptosis and necrosis and suppression of oxidative stress are the main signaling pathways to be considered [69].

We also observed hypermetabolism in the bed nucleus of the stria terminalis, which has been shown to be associated with anxiety [70] and with behavioral responses to unfamiliar things [71]. However, we are not sure whether this is caused by the fear of acupuncture, and this postulation still needs to be further confirmed. In our study, we chose distant nonacupoints mainly because the rat's head was too small, so needling at completely different sites could exert a better perspective of the brain regional effects.

There are other limitations in this study: (1) the AD model was induced by the intraperitoneal injection of D-gal and the bilateral hippocampus injection of A*β*1–40, which could mimic some symptoms of AD, but it was difficult to fully demonstrate the characteristics of AD patients. This model was established according to the previous studies [72–74]. (2) We simply used the Y-maze to test the progression of AD rats, but it may be better for us to analyze some biochemical markers at the same time. (3) We were unable to explain why acupuncture did not target the corresponding pathological brain areas in AD rats. Future research should also explore the relationship between the brain areas in AD rats that exhibit hypometabolism and the brain areas where acupuncture activates hypermetabolism.

## 5. Conclusion

This study demonstrated that acupuncture at GV24 and bilateral GB13 acupoints could improve learning and memory abilities of the AD rats and increase the CGM in the thalamus, hypothalamus, and brain stem. These findings suggest that the neuromodulatory mechanism of acupuncture may be achieved by enhancing synaptic plasticity and regulating the production of certain kinds of neurotransmitters, but longitudinal studies might be needed to confirm this explanation. These preclinical findings will undoubtedly enhance the understanding of the pathophysiological mechanisms of AD. The improved perspectives on brain functional neurocognition will be helpful in understanding the mechanism of acupuncture and may also serve as a fundamental basis to diagnose and develop efficient treatment to improve the quality of life of AD patients and their families.

## Figures and Tables

**Table 1:** Reagents and instruments.

Reagents and instruments	Source
D-Galactose	Shanghai No. 2 Reagent Company, China
A*β*1–40	Sigma Co. Ltd., USA
Sodium pentobarbital	Beijing Chemical Reagents Company, China
Sodium benzylpenicillin	Harbin Pharmaceutical Group Co. Ltd., China
Sterile normal saline	Guangdong Dazhong Pharmaceutical Co. Ltd., China
Isoflurane	Hebei Jiupai Pharmaceutical Co. Ltd., China
Stereotaxic apparatus	Xi'an Northwest Photoelectric Instrument Factory, China
MicroPET imaging system	Siemens, Germany
ECAT EXACT HR + PET imaging system	Siemens, Germany
Matrx animal anaesthesia machine	Midmark Corporation, USA
Y-Maze	Yuanyang Zhenhua Teaching Instrument, Hebei, China
Acupuncture needles	Suzhou Acupuncture & Moxibustion Appliance Co. Ltd., China

**Table 2:** Areas with higher PET signals (control group versus model group).

Area	Size (voxels)	Local maximum *t* value	Talairach coordinates (mm)			Side	Related brain region	Panel in Figure 3(a)
			*x*	*y*	*z*			
1	8804	7.27	1.64	0.47	−3.48	Right	Parietal association cortex	A1
2	8804	7.81	0.49	1.51	2.28	Right	Cingulate gyrus	A1
3	8804	8.51	1.80	0.63	−5.16	Right	Visual cortex	A1
4	8804	8.19	−0.22	0.41	−3.96	Left	Retrosplenial cortex	A2
5	1733	5.63	0.88	3.63	3.24	Right	Prelimbic cortex	A3
6	1733	4.87	2.50	6.15	2.28	Right	Striatum	A3
7	1733	6.47	3.15	4.77	3.48	Right	Insular cortex	A4
8	1733	6.21	2.88	4.75	1733	Right	Orbital cortex	A4
9	605	8.48	0.83	3.88	7.08	Right	Olfactory bulb	A1

**Table 3:** Areas with higher PET signals (acupoint group versus model group).

Area	Size (voxels)	Local maximum *t* value	Talairach coordinates (mm)			Side	Related brain region	Panel in Figure 3(b)
			*x*	*y*	*z*			
1	1608	3.93	−1.20	6.21	−0.84	Left	Bed nucleus of stria terminalis	B1
2	1608	4.84	0.29	6.59	−2.52	Right	Dorsal thalamus-lateral nucleus group	B1
3	1608	5.85	0.03	7.03	−2.76	Right	Dorsal thalamus-midline nucleus group	B1
4	1608	5.34	0.03	7.22	−3.24	Right	Hypothalamus-mammillary region	B1
5	1608	3.71	0.28	7.57	−2.04	Right	Hypothalamus-supraoptic region	B1
6	1608	5.24	−0.24	7.58	−2.52	Left	Hypothalamus-tuberal region	B1
7	482	4.50	3.65	6.60	−3.72	Right	Midbrain-cerebral peduncle	B2
8	482	3.76	0.70	7.26	−3.24	Right	Midbrain tegmentum	B2
9	259	3.99	1.22	7.44	−11.64	Right	Pontine tegmentum	B1

**Table 4:** Areas with higher PET signals (nonacupoint group versus model group).

Area	Size (voxels)	Local maximum *t* value	Talairach coordinates (mm)			Side	Related brain region	Panel in Figure 3(c)
			*x*	*y*	*z*			
1	172	4.99	0.16	7.06	−3.00	Right	Dorsal thalamus-midline nucleus group	C1
2	172	4.64	0.17	7.23	−3.24	Right	Hypothalamus-mammillary region	C1
3	172	4.14	0.30	7.51	−3.00	Right	Hypothalamus-tuberal region	C1
4	108	3.97	−3.19	5.62	−2.04	Left	Dorsal thalamus-lateral nucleus group	C2
